# Fluid creep in burn resuscitation: the tide has not yet turned

**DOI:** 10.1186/cc11071

**Published:** 2012-03-20

**Authors:** E James, M Hayes, P McCabe, G Williams, M Takata, MP Vizcaychipi

**Affiliations:** 1 Chelsea and Westminster Hospital and Imperial College, London, UK

## Introduction

The purpose of this study was to examine the fluid resuscitation of severely burned patients admitted to our regional centre and to review whether our practice had changed over the last 5 years in light of concerns of fluid creep. Fluid creep is the term coined by Pruitt used to describe fluid resuscitation in excess of that predicted by the Parkland formula and which is associated with abdominal compartment syndrome (ACS) [1].

## Methods

We completed a retrospective review in accordance with clinical governance guidance of patient notes evaluating all admissions in two groups (Group A: 1 May 2005 to 30 April 2006 and Group B: 1 May 2010 to 30 April 2011). The review examined the first 72 hours of fluid resuscitation in patients with ≥15%TBSA burns who were admitted less than 24 hours post burn injury.

## Results

There were 12 patients in each group. Both groups were comparable in both admission (Table 1) and resuscitation data. The total fluid (mean ± SD) given in the first 24 hours post burn-centre admission was 5.36 ± 2.22 ml/kg/%TBSA in Group A and 5.72 ± 3.00 ml/kg/%TBSA in Group B (*P *= 0.817) with three patients in each group receiving in excess of 250 ml/kg. Almost one-third of the fluid administered was colloid in each group. The hourly urine output (mean ± SD) was 1.34 ± 0.72 ml/kg/hour in Group A and 1.53 ± 1.47 ml/kg/hour in Group B (*P *= 0.817). Inhalational injury was present in six patients in Group A and three in Group B. The inhalational injury group (mean ± SD) received 6.64 ± 2.51 ml/kg/%TBSA whilst the noninhalational injury group received 4.88 ± 2.50 ml/kg/%TBSA (*P *= 0.101). There was no reported incidence of ACS.

**Table 1:** 

Patient data	Group A	Group B	*P *value
Number (*n*)	12	12	
Age (years)	49 (18 to 69)	38.5 (21 to 77)	0.260
Weight (kg)	72 (55 to 109)	75 (60 to 99)	0.794
% TBSA	37.5 (16 to 70)	31 (18 to 60)	0.602
Inhalation injury (*n*)	6/12	3/12	0.206
Trauma (*n*)	1/12	0/12	0.307
Admission base defi cit	-5.95 (-15 to +1)	-6.55 (-11.7 to +2.5)	0.931
Admission lactate (mmol/l)	3.03 (0.98 to 5.4)	2.05 (0.5 to 4.1)	0.081
Survival (*n*)	6/12	9/12	0.206

Data presented as median (range).

## Conclusion

Despite our awareness of fluid creep, our practice has not changed significantly over the last 5 years. Fluid was administered in excess of that predicted by the Parkland formula despite almost one-third being given as colloid and no cases of ACS being reported. A multicentre randomised control trial is required to examine stricter titration of fluid administration to urine output and the specific role of colloids in early resuscitation.
